# Emergence of OXA-48-Producing *Klebsiella pneumoniae* in Taiwan

**DOI:** 10.1371/journal.pone.0139152

**Published:** 2015-09-28

**Authors:** Ling Ma, Jann-Tay Wang, Tsu-Lan Wu, L. Kristopher Siu, Yin-Ching Chuang, Jung-Chung Lin, Min-Chi Lu, Po-Liang Lu

**Affiliations:** 1 National Institutes of Infectious Diseases and Vaccinology, National Health Research Institutes, Miaoli, Taiwan; 2 Department of Internal Medicine, National Taiwan University Hospital, Taipei, Taiwan; 3 Department of Clinical Pathology, Linkou Chang Gung Memorial Hospital, Taoyuan, Taiwan; 4 Department of Internal Medicine and Medical Research, Chi Mei Medical Center, Tainan, Taiwan; 5 Department of Internal Medicine, Chi Mei Medical Center, Liouying, Tainan, Taiwan; 6 Division of Infectious Diseases and Tropical Medicine, Department of Internal Medicine, Tri-Service General Hospital, National Defense Medical Center, Taipei, Taiwan; 7 Section of Infectious Diseases, Department of Medicine, Chung Shan Medical University Hospital, Taichung, Taiwan; 8 Department of Internal Medicine, Kaohsiung Medical University Hospital, Kaohsiung, Taiwan; 9 College of Medicine, Kaohsiung Medical University, Kaohsiung, Taiwan; University of Pittsburgh, UNITED STATES

## Abstract

The isolation of OXA-48-producing *Enterobacteriaceae* has increased dramatically in Mediterranean countries in the past 10 years, and has recently emerged in Asia. Between January 2012 and May 2014, a total of 760 carbapenem non-susceptible *Klebsiella pneumoniae* (CnSKP) isolates were collected during a Taiwan national surveillance. Carbapenemases were detected in 210 CnSKP isolates (27.6%), including 162 KPC-2 (n = 1), KPC-3, KPC-17, and NDM-1 (n = 1 each), OXA-48 (n = 4), IMP-8 (n = 18), and VIM-1 (n = 24). The four *bla*_OXA-48_ CnSKP isolates were detected in late 2013. Herein we report the emergence OXA-48-producing *K*. *pneumoniae* isolates in Taiwan. PFGE analysis revealed that the four isolates belonged to three different pulsotypes. Three isolates harboured *bla*_CTX-M_ genes and belonged to MLST type ST11. In addition, the plasmids belonged to the incompatibility group, IncA/C. One isolate belonged to ST116 and the plasmid incompatibility group was non-typeable. The sequence upstream of the *bla*_OXA-48_ gene in all four isolates was identical to pKP_OXA_-48N1, a *bla*_OXA-48_-carrying plasmid. This is the first report of OXA-48-producing *Enterobacteriaceae* in Taiwan and the second report to identify *bla*_OXA-48_ on an IncA/C plasmid in *K*. *pneumoniae*. Given that three isolates belong to the same pandemic clone (ST11) and possess the IncA/C plasmid and similar plasmid digestion profile that indicated the role of clonal spread or plasmid for dissemination of *bla*_OXA-48_ gene, the emergence of OXA-48-producing *K*. *pneumoniae* in Taiwan is of great concern.

## Background

Different from the third and fourth generations of cephalosporins, carbapenems are currently more consistently active against *Enterobacteriaceae* because carbapenems are not inactivated by extended-spectrum β-lactamases (ESBLs) or plasmid-encoded AmpC cephalosporinases. Resistance to carbapenems is relatively rare; therefore, this class of drugs is considered the last option for the treatment of severe infections.

Carbapenemases are described as chromosomally-encoded β-lactamases prior to the identification of plasmid-encoded IMP-1, OXA-23 (ARI-1), and KPC-2. The emergence of plasmids containing carbapenemase genes, including KPC-type (class A), as well as IMP-,VIM-, and NDM-types (class B), is considered a serious threat as the emergence of plasmids containing carbapenemase genes facilitates the dissemination of carbapenem resistance [1], and the carbapenemases hydrolyze almost all β-lactams. Indeed, plasmid-borne carbapenemases have been isolated worldwide, including Taiwan [1–4]. After the first identification of a *Klebsiella pneumoniae* strain expressing plasmid-encoded class D carbapenemase OXA-48 in Istanbul [5], the incidence of OXA-48 expression by *Enterobacteriaceae* has increased dramatically in Mediterranean countries [6]. In 2012, a 4-year surveillance program was initiated in Taiwan to investigate carbapenem resistance mechanisms, especially with respect to plasmid-borne carbapenemases in *K*. *pneumoniae* and *Escherichia coli*. Herein we report the identification of the first four isolates expressing OXA-48 during this surveillance program.

## Methods

### Bacterial strains and susceptibility testing

The participating hospitals in the national survey identified imipenem or meropenem non-susceptible *K*. *pneumoniae* and *E*. *coli* isolates and sent the isolates to a reference laboratory at the National Health Research Institutes of Taiwan. A total of 760 imipenem- or meropenem-non-susceptible *K*. *pneumoniae* and 144 imipenem- or meropenem-non-susceptible *E*. *coli* isolates were consecutively collected from 18 hospitals (11 medical centers and 7 regional hospitals) between January 2012 and April 2014. The participating hospitals in the surveillance program distributed in all regions of the country. The regional hospitals can handle most diseases and injuries; however, medical centers provide services with more specialists, especially critical care specialist and specialists who can diagnose and treat immune-compromised patients. All the isolates were from individual cases.

This study was approved by the Institutional Review Boards of all participating hospitals, including Kaohsiung Medical University Chung-Ho Memorial Hospital (KMUH-IRB-20110328), Linkou Chang Gung Memorial Hospital (1003399B), Chung Shan Medical University Hospital (CSMUH-CS-12187), and National Taiwan University Hospital (201110043RB).

The isolates were obtained as part of routine hospital care procedures, and written informed consent for participation in the study was waived. The primary screening for carbapenem resistance was performed by the individual participating hospitals. Further confirmation of antimicrobial susceptibility was determined using the broth micro-dilution method according to the guidelines of the Clinical and Laboratory Standards Institute (CLSI) [7].

Minimum inhibitory concentrations (MICs) for carbapenems (ertapenem, imipenem, meropenem, and doripenem) and other antimicrobial agents, including cefazolin, cefotaxime, cefoxitin, cefepime, ciprofloxacin, amikacin, gentamicin, trimethoprim-sulfamethoxazole (SXT), colistin, and tigecycline, were determined using the broth micro-dilution method (Sensititre; Trek Diagnostic Systems, Cleveland, OH, USA). CLSI M100-S24 [8] interpretive breakpoints were used to interpret the MIC results for all antimicrobial agents studied, with the exception of tigecycline and colistin. The Food and Drug Administration (FDA) breakpoint was used for tigecycline and the European Committee on Antimicrobial Susceptibility Testing (EUCAST) breakpoint was used for colistin.

### Detection of genes encoding carbapenemases, AmpC, and ESBLs

Carbapenemase genes (e.g., class B families [*bla*_IMP_, *bla*_VIM_, *bla*_NDM_, *bla*_GIM_, *bla*_SPM_, and *bla*_SIM_], class A families [*bla*_NMC_, *bla*_IMI_, *bla*_SME_, *bla*_KPC_, and *bla*_GES_], and class D [*bla*_OXA-48_]), plasmid-encoding AmpC (e.g., *bla*_CMY_, *bla*_DHA_, *bla*_FOX_, and *bla*_ACT_) [9], and ESBL genes (e.g., *bla*_CTX-M_, *bla*_TEM_, and *bla*_SHV_) were detected by PCR amplification [4]. The amplicons were sequenced, and the entire sequence of each gene was compared to the sequences in the GenBank nucleotide database at http://www.ncbi.nlm.nih.gov/blast/.

### Characteristics of *bla*_OXA-48_-containing plasmids

Plasmid conjugation was performed using *E*. *coli* J53 AzR as the recipient strain. The recipients and *bla*_OXA-48_–carrying donor samples were separately inoculated into brain-heart infusion broth and incubated at 37°C for 4 h. The samples were then mixed at a ratio of 10:1 (donor: recipient [*v*: *v*]) for an overnight incubation at 37°C. A 0.1-mL aliquot of the overnight broth mixture was spread onto a MacConkey agar plate containing sodium azide (100 μg/mL) and imipenem (1 μg/mL). The plasmids were extracted from these conjugants using a standard alkaline lysis method, and the fingerprints of the plasmids were generated by digestion with *Eco*RI (New England Biolabs, Beverly, MA, USA), as previously described [10]. Plasmid incompatibility was determined by a PCR-based replicon typing scheme, as previously described [11]. PCR and sequencing of the upstream of *bla*_OXA-48_ gene were performed using the following primers in this study: OXA-48F, 5’-CGCATCTTGTTGTCCAAGTG-3’; and OXA-48R, 5’-TCGAGCATCAGCATTTTGTC-3’. The full sequence length was 1012 bp.

### Pulsed-field gel electrophoresis (PFGE)

Total DNA was prepared and digested with *Xba*I (New England Biolabs), as suggested by the manufacturer. The restriction fragments were separated by PFGE in a 1% agarose gel (Bio-Rad, Hercules, CA, USA) with 0.5× TBE buffer (45mM Tris, 45mM boric acid, and 1.0mM EDTA [pH 8.0]) for 22 h at 200 V and 14°C, with ramp times of 2–40 s using a CHEF Mapper apparatus (Bio-Rad Laboratories, Richmond, CA, USA). The Dice coefficient was used to calculate similarities, and the unweighted pair-group method with the arithmetic mean (UPGMA) was used for the cluster analysis using the BioNumerics software (version 5.10; Applied Maths, St-Martens-Latem, Belgium).

### Multi-locus Sequence Type

Multi-locus sequence typing (MLST) was performed on the four OXA-48-positive *K*. *pneumoniae* isolates according to the protocol described on the *K*. *pneumoniae* MLST website (http://www.pasteur.fr/recherche/genopole/PF8/mlst/Kpneumonia.html). MLST results were typed according to the international *K*. *pneumoniae* MLST database, which was created in 2005 at the Pasteur Institute in Paris, France.

## Results

### An overall description of the findings in the surveillance program

Of 760 carbapenem non-susceptible *K*. *pneumoniae* (CnSKP) isolates, the resistant rates to imipenem, meropenem, doripenem, and ertapenem were 74.7%, 68.3%, 67.3%, and 92.3%, respectively. Carbapenemases were detected in 210 CnSKP isolate (27.6%), including 162 KPC-2, 4 OXA-48, 18 IMP-8, 24 VIM-1, and 1 each of KPC-3, KPC-17, and NDM-1. Almost all KPC-2 CnSKP isolates had the same PFGE type and belonged to MLST ST11. The four *bla*_OXA-48_ CnSKP isolates were detected in late 2013.

### Case descriptions

None of the patients had a travel history abroad. Isolates 1 and 2 were acquired in one hospital in central Taiwan. The isolates possessed the same genes, including *bla*_OXA-48_, *bla*_CTX-M-14_, *bla*_TEM-31_, and *bla*_SHV-11_. Patient 1 was an 85-year-old female who suffered from pneumonia with poorly controlled type 2 diabetes. She was admitted to another hospital before being transferred. The use of antibiotics was not known. Patient 2 was a 78-year-old female who had sepsis, hospital-acquired pneumonia, and a catheter-related infection. The patient was treated with ertapenem for 7 days prior to isolation of the resistant strain. Patients 1 and 2 had overlapping stays in the same unit, which suggested an epidemiologic relationship between cases 1 and 2. Isolates 3 and 4 were isolated from two different medical centers within the same city in northern Taiwan; however, there were no patient transfers between these two centers for the two cases. Isolate 3, which carried *bla*_OXA-48_, *bla*_CTX-M-15_, *bla*_TEM-1_, and *bla*_SHV-11_, was obtained from an 82-year-old hospitalized patient who had pneumonia and was transferred from another hospital. Isolate 4 was obtained from a 56-year-old outpatient who had a toothache and had received treatment for dental caries in a community clinic. Isolate 4 had no other extended-spectrum cephalosporinase genes and no permeability defects; isolate 4 was susceptible to the late generation cephalosporins, but resistant to carbapenems. No epidemiologic linkage was found between cases in northern and central Taiwan.

### Susceptibilities and extended spectrum β-lactamases of four OXA-48 producers

The antimicrobial susceptibility pattern and the β-lactamases genes carried by the four *K*. *pneumoniae* isolates carrying *bla*_OXA-48_ are presented in Table 1. Isolate 4 (from case 4) harboured *bla*_OXA-48_ alone and was resistant to ampicillin, cefazolin, piperacillin-tazobactam, ticarcillin-clavulanate, ertapenem, imipenem, meropenem, and doripenem. Isolate 4 had intermediate resistance to cefuroxime and was susceptible to cefotaxime, ceftazidime, cefepime, aztreonam, cefoxitin, gentamicin, amikacin, nalidixic acid, ciprofloxacin, trimethoprim-sulfamethoxazole, colistin and tigecycline (Table 1). Isolates 1 and 2 harboured *bla*_OXA-48_, *bla*_CTX-M-14_, and *bla*_TEM-31_, and were resistant to all of the above antibiotics except colistin and tigecycline. Isolate 3 encoded *bla*_OXA-48_ and *bla*_CTX-M-15_, and was resistant to all of the above antibiotics, except tigecycline (Table 1).

**Table 1:** Genetic features of four *bla*_OXA-48_ *Klebsiella pneumoniae*.

Isolate	Specimen	β-lactam MICs (μg/mL)					ST type	Non-β-lactamassociated resistance	Associated β-lactamases	Inc
		ERT	IMP	MEM	CAZ	CTX				
1	sputum	≥8	≥8	≥8	≥32	≥64	11	Gm, Ak, Q, SXT	CTX-M-14, TEM-31, SHV-11	IncA/C
2	urine	≥8	≥8	≥8	16	≥64	11	Gm, Ak, Q, SXT	CTX-M-14, TEM-31, SHV-11	IncA/C
3	urine	≥8	≥8	≥8	≥32	≥64	11	Gm, Ak, Q, SXT, Cs	CTX-M-15, TEM-1, SHV-11	IncA/C
4	urine	≥8	≥8	4	≤1	≤1	116	none	SHV-1	NT

ERT: ertapenem; IMP: imipenem; MER: meropenem; CAZ: ceftazidime; CTX: ceftaxime; Gm: gentamicin; Ak: amikacin; Cs: colistin; MIC: minimum inhibitory concentration; SXT: trimethoprim-sulfamethoxazole; Q: fluoroquinolones; NT: not-typeable

### Plasmid digestion profile

**Fig 1:**
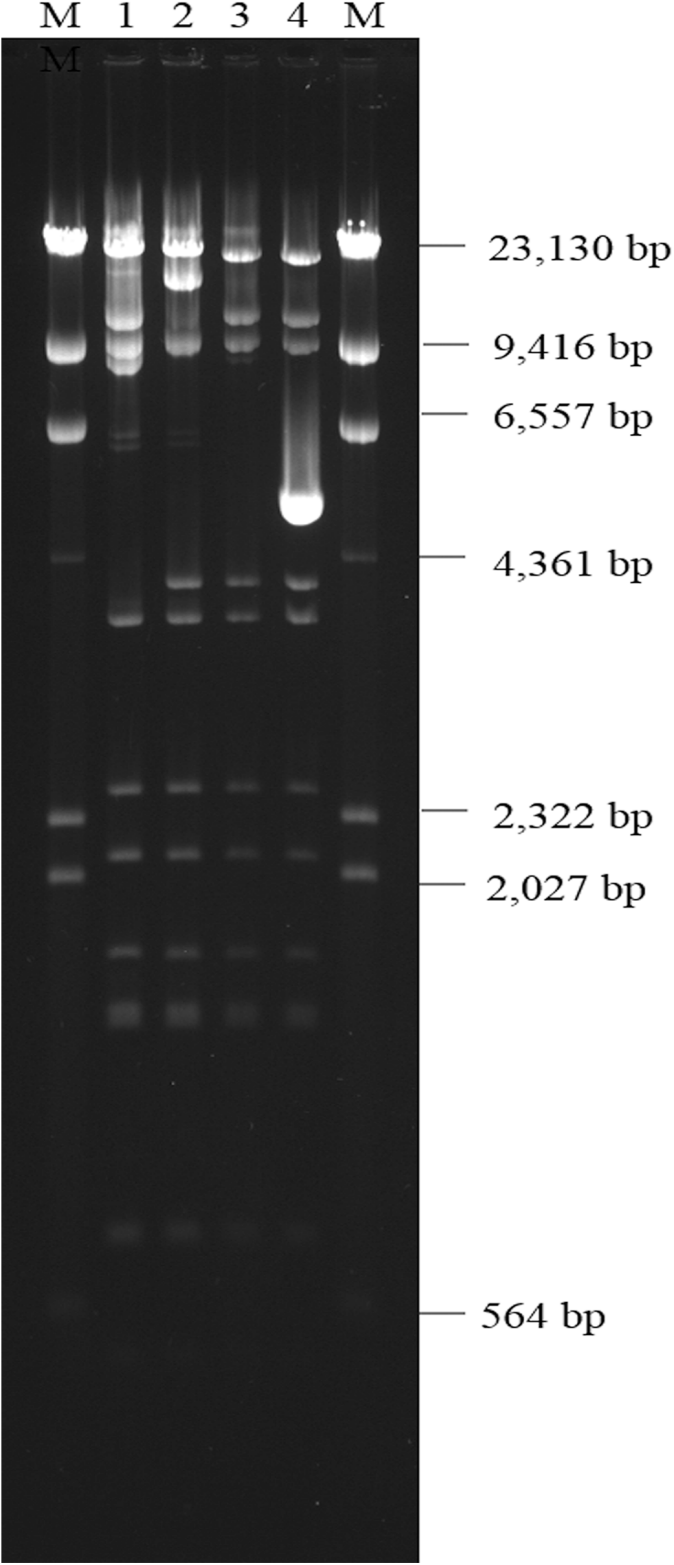
EcoRI-digested plasmid digestion profile of four OXA-48-producing *Klebsiella pneumoniae* isolates. M, marker.

Molecular analysis with PCR and plasmid extraction of the *E*. *coli* conjugants revealed that *bla*_OXA-48_ and *bla*_CTX-M_ were detected on the same plasmid in isolates 1–3. The plasmid belongs to the incompatibility group, IncA/C (Table 1). The incompatibility group of the plasmid from isolate 4 was not typeable by the PCR-based replicon typing method. The plasmids of conjugants of isolates 1, 2, and 3 exhibited similar restriction profiles (*Eco*RI digested; Fig 1). The upstream (950 bp) of all 4 plasmids were identical to pKP_OXA_-48N1 (GenBank database accession number KC757416) [12].

### PFGE and MLST analyses

**Fig 2:**
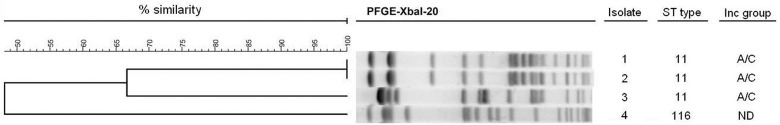
Dendrogram of XbaI-digested genomic DNA of four OXA-48 producing *Klebsiella pneumoniae* isolates.

As shown in Fig 2, PFGE revealed 3 clones; isolates 1 and 2 were the same clone and distinct from isolates 3 and 4. Analysis of the ST type revealed that isolates 1–3 belong to ST11, and isolate 4 belongs to ST116 (Table 1 and Fig 2).

## Discussion

Because *bla*_OXA-48_ was first detected in *K*. *pneumoniae* in Turkey [5], it has spread rapidly throughout the Middle East and subsequently in Europe, North Africa, North America, and Asia. Most OXA-48-producing isolates were *K*. *pneumoniae*; however, OXA-48 was also identified in other *Enterobacteriaceae*, such as *E*. *coli*, *Enterobacter* spp., *Klebsiella oxytoca*, *Citrobacter freundii*, and *Serratia marcecens* [13].

This is the first report of OXA-48-producing *K*. *pneumoniae* in Taiwan. All four isolates appeared in late 2013, but belonged to three different pulsotypes. Moreover, with the exception of cases 1 and 2, no epidemiologic linkage was identified between the 4 cases. The absence of *bla*_OXA-48_ detection in the national surveillance program from 2012–2013 suggests that *bla*_OXA-48_ is a new resistance gene; however, the epidemiologic data did not reveal that this carbapenemase gene was transmitted from other countries.

Previous studies reported that most *bla*_OXA-48_ genes were located on a 62 kb self-conjugative plasmid in *Enterobacteriaceae* (pOXA-48a from the *K*. *pneumoniae* 11978 isolate from Turkey or plasmid pKP_OXA_-48N1) [12]. In 2013, a new 160-kb *bla*_OXA-48_-encoding conjugative plasmid, pKP_OXA_-48N2, was characterized from *K*. *pneumoniae* [12]. The flanking region containing *bla*_OXA-48_ in Taiwan isolates was identical to that observed in pKP_OXA_-48N1 [12]. Comparative analysis of the upstream region of three kinds of *bla*_OXA-48_-carrying plasmids (pOXA-48a, pKP_OXA_-48N1, and our *bla*_OXA-48_ plasmids) revealed that the plasmids have identical 164 bp sequences before the *bla*_OXA-48_ start codon, including IS*1999* insertion sequences. Further upstream of this 164 bp sequence was a difference between pOXA-48a and pKP_OXA_-48N1. The sequence of our *bla*_OXA-48_ carrying plasmids at approximately 950 bp upstream was identical to pKP_OXA_-48N1. Moreover, three plasmids exhibited very similar restriction profiles (*Eco*RI) over 60-kb, and matched the predicted digestion profile of pKP_OXA_-48N1. The plasmid digestion profile of that obtained from isolate 4 also had a similar restriction profile with the exception of containing one additional fragment of approximately 5-Kb. The results indicate the role of plasmids in the dissemination of *bla*_OXA-48_. Approximately 80% of OXA-48 producers co-expressed ESBLs [14]; many OXA-48-producing isolates also carried CTX-M-15 [14–16]. In our OXA-48-producing isolates, three isolates also expressed CTX-M enzymes. According to previous surveillance data, the IncA/C-type plasmid was associated with *bla*_CTX-M-15_(12.7%) in Asian countries [17]. In this study, three *bla*_OXA-48-_containing plasmids also harboured *bla*_CTX-M_ genes and belonged to the IncA/C group. Although most *bla*_CTX-M-15_ were associated with the IncFIIA plasmid, the IncA/C plasmid has also been shown to be related with *bla*_CTX-_M, and presumably the dissemination of CTX-M enzymes [17, 18]. Unlike the epidemic *bla*_OXA-48_ plasmid incompatibility type, IncL/M [19], this is the second study to identify *bla*_OXA-48_ on an IncA/C plasmid [20].

In an 11-year (2001–2011) molecular epidemiologic study of OXA-48 in Europe and North Africa, ST101 was the most frequently observed, followed by ST395 and ST15 in *K*. *pneumoniae*; ST11 was rarely detected [16]. In other regions, however, ST11 was the most prevalent ST type for multi-resistant *K*. *pneumoniae* [21]. OXA-48-producing isolates have been reported in Turkey, Argentina, and France [15,22]. In Greece, an OXA-48-producing ST11 clone caused an outbreak [23]. With the exception of one isolate (ST116), three of the four OXA-48 producers identified in Taiwan belonged to ST11, the predominant sequence type outside Europe and North Africa. ST11 has been found in association with different ESBLs, primarily CTX-M-15, CTX-M-14, and SHV-5 [24, 25]. ST11 is also the predominant sequence type of imipenem non-susceptible *K*. *pneumoniae* isolates with resistance mechanisms other than OXA-48 in Taiwan [3]. Our report identified ST11 to be the major sequence type for OXA-48 producers.

In conclusion, we identified the first cluster of OXA-48 carbapenem-resistant *K*. *pneumoniae* in Taiwan. Although identification of OXA-48 producers is still sporadic in Asia, studies have predicted that OXA-group enzymes will successfully spread in the near future [26]. The association between OXA-48 and CTX-M could potentially lead to pan-β-lactam resistance [14]. Considering the high prevalence of ST11 *K*. *pneumoniae* and incorporation into the IncA/C plasmid with pandemic CTX-M enzymes, the emergence of OXA-48 is of great concern.
