# Correction: Emergence of OXA-48-Producing *Klebsiella pneumoniae* in Taiwan

**DOI:** 10.1371/journal.pone.0141179

**Published:** 2015-10-20

**Authors:** The PLOS ONE Staff

## Notice of Republication

The original S1 File was published in error. The file was removed from the HTML and PDF versions of this article on October 1, 2015.
